# Some Delusions Regarding the Oyster

**Published:** 1883-03

**Authors:** Charles L. Dana

**Affiliations:** New York City


SOME DELUSIONS REGARDING THE OYSTER. By CHARLES L. DANA, M.D., of New York City.

The oyster does not present a very lofty theme, and I venture to apologize first for calling attention to it at the present length. Mr. Herbert Spencer, in his address upon America, made no reference to this interesting animal, and we are left to infer that it has no importance in American society, and no definite relation to the problems of evolution. But one may fairly claim that this is a neglect, and that he went too far in ignoring what is so unobtrusive. For the oyster represents, very typically, that absence from work and worry which should characterize the evolved life toward which Americans are advised to strive. Furthermore, the oyster, besides thus offering us certain valuable ideals, is a very considerable factor in the social life of the "R" months.

With this preamble, I venture to submit some corrections of prevalent errors regarding the mollusk in question.

1. That the oyster digests itself. For several years the statement, quite uncontradicted, has been going the rounds of the press, that the oyster digests itself. I believe that Dr. Wm. Roberts first gave currency to it. The theory is that the oyster has a large liver, which contains a diastase, and that this diastase, in some inscrutable way, digests the whole animal, under suitable conditions. Thus it has become a wide-spread belief that the oyster, taken into the stomach, does, by virtue of its liver, execute a kind of *felo de se.* Such a belief is very consoling when a person is committing midnight indiscretions with

*ostrea edalis*, and it is unpleasant to be obliged to dispel it. Yet it is a fact, which the accompanying record of experiments will show, that an oyster has no more self-digestive power than a man.

The hepatic disatase referred to has no power except to change glycogen into sugar—a very trivial matter. It cannot even digest the liver tissue. I have kept oysters, previously crushed between the teeth, in water (temperature 100° F.) acidulated, and neutral for hours, with no resulting digestion whatever.) See experiments iii., iv., v.) I have even dissected out the liver, and given it the best possible chance to eat itself; but neither the mystic diastase nor any other ferment at all affected its succulent autonomy. The oyster does not and cannot digest itself.

2. That raw oysters are always more digestible than the cooked. I quite admit that the ordinary stew is less digestible than the plate of raw oysters. The stew generally contains milk, butter, and a larger number of oysters, all of which complicates the question. Half a dozen oysters, however, roasted* in the shell, or simply boiled a short time, will be digested nearly if not quite as rapidly as the same number of raw (See exper. i., ii). Cooking coagulates the albumen, but coagulated albumen may be more digestible than raw. Thus the white of an egg, unless thoroughly beaten, is lowly digested, and similarly, raw beef has to be finely minced in order to be quickly affected by the gastric juice. Cooking, on the other hand, loosens the tissue binding together the muscular fibrils, and allows the peptic juices to penetrate.

3. That fermented liquors dissolve or digest the oyster. Currency has been given in the *Reporter* and many other journals to the following highly instructive tale: Rev. Dr. Houghton, of Dublin, clergyman, physician and physiologist, was sitting with a friend at a restaurant. Raw oysters had been brought them. Believing, however, that it is proper *desipere in loco*, Dr. Houghton's friend ordered brandy; he himself ordered ale. Wishing to demonstrate the wisdom of his choice and the beauty of physiological processes, Dr. Houghton poured some brandy into one glass, and ale into another, and then dropped an oyster into each. The oyster in the brandy grew hard and shrivelled; that in the

* Beaumont found the difference in his single case to be only 20 minutes.

ale gradually melted away in a diffusible invisible solution. *Moral:* Drink ale with oysters.

Now, Dr. Houghton's name and authority have great weight. I doubt if the incident related really occurred, yet it is widely circulated and credited. But it is quite as well, if one is bound to have bad and bibulous habits, to put them upon as near as possible a physiological basis. Therefore I venture to deny the possibility and accuracy of Dr. Houghton's alleged experiment, at least as regards American oysters. These grow hard in ale or beer, instead of dissolving. (Experiment vi.)

When any one becomes so dissolute, therefore, as to drink fermented liquors with his oysters, he should not allow his habits to be confirmed by a false confidence in the potency of malt diastase.

The following is a brief record of experiments made:

| OYSTER. | TEST SOLUTION. | TEMP. | DURATION OF EXPERIMENT. | RESULT. |
|---|---|---|---|---|
| I. Raw, masticated. | Aqæ, ℥ iij. HCl. 1 per cent. Pepsin (pure) gr. v. | 100° F. to 110° F. | 3½ hours. | Almost dissolved. Fine fragments and a piece of adduct or muscle left. |
| II. Boiled for 30 seconds. | Same solution as above. | As above. | As above. | About same result. Possible a few more fine fragmet's. |
| III. Raw, masticated. | Aquæ, ℥ iij. | As above. | As above. | Little if any apparent change. Liver not affected. |
| IV. Raw, masticated. | Aquæ, ℥iij. Sodæ bicarb, gr. x. | As above. | As above. | Same result apparently as in III. |
| V. Raw, masticated. | Aquæ, ℥ iij. HCl., 1 per cent. | As above. | As above. | Oyster tissue much softened; liver nearly gone. |
| VI. Raw, cut into once to expose liver. | Beer, ℥ viij. | As above. | 4 hours. | Oyster hardened; liver not appreciably affected. |
| VII. Raw, cut into once as above. | Aquæ, ℥ ijss. Brand, ℥ ss. or 6 per cent. | As above. | 4 hours. | Oyster tissue a little softer. |

—*Medical and Surgical Reporter.*

